# Genetic × environment variation in sheep lines bred for divergent resistance to strongyle infection

**DOI:** 10.1111/eva.13294

**Published:** 2021-09-15

**Authors:** Guillaume Sallé, Véronique Deiss, Céline Marquis, Gwenola Tosser‐Klopp, Jacques Cortet, Delphine Serreau, Christine Koch, Didier Marcon, Frédéric Bouvier, Philippe Jacquiet, Alexandra Blanchard, Marie‐Madeleine Mialon, Carole Moreno‐Romieux

**Affiliations:** ^1^ INRAE UMR1282 ISP U. de Tours Nouzilly France; ^2^ INRAE VetAgro Sup UMRH F‐63122 Saint‐Genès‐Champanelle U. Clermont Auvergne Theix France; ^3^ UE332 La Sapinière INRAE Osmoy France; ^4^ INRAE ENVT UMR1388 GENPHYSE U. de Toulouse Castanet‐Tolosan France; ^5^ ENVT UMR1225 IHAP INRAE Toulouse France; ^6^ Pancosma SA Geneva Switzerland

**Keywords:** environment, G × E, nematode, resistance, sheep, SNP, stress

## Abstract

Drug‐resistant parasites threaten livestock production. Breeding more resistant hosts could be a sustainable control strategy. Environmental variation linked to animal management practices or to parasite species turnover across farms may however alter the expression of genetic potential. We created sheep lines with high or low resistance to *Haemonchus contortus* and achieved significant divergence on both phenotypic and genetic scales. We exposed both lines to chronic stress or to the infection by another parasite *Trichostrongylus colubriformis*, to test for genotype‐by‐environment and genotype‐by‐parasite species interactions respectively. Between‐line divergence remained significant following chronic stress exposure although between‐family variation was found. Significant genotype‐by‐parasite interaction was found although *H. contortus*‐resistant lambs remained more resistant against *T. colubriformis*. Growth curves were not altered by the selection process although resistant lambs were lighter after the second round of divergence, before any infection took place. Breeding for resistance is a sustainable strategy but allowance needs to be made for environmental perturbations and worm species.

## 1 INTRODUCTION

Gastro‐intestinal nematodes (GIN) impose a significant burden to human health and livestock worldwide. Repeated systematic anthelmintic drug treatments have favoured the rapid selection of drug‐resistant isolates across continents (Kaplan & Vidyashankar, 2012), rendering sheep farming impossible in some cases (Blake & Coles, 2007). Concerns about environmental side effects associated with anthelmintic drug metabolites (Verdú et al., 2018) have also driven research efforts to develop alternative control strategies.

Breeding more resistant individuals is a promising alternative. Indeed, domestic (Bishop, 2012; Gruner et al., 2004; Woolaston, 1992) and wild populations (Gold et al., 2019; Smith et al., 1999; Sparks et al., 2019) often show heritable genetic variation for resistance to parasite infection that breeding programmes could exploit. This trait has a polygenic architecture. This has been described with genome‐wide resolution in commercial sheep populations (Kemper et al., 2011) and evidence of multiple Quantitative Trait Loci (QTL) with mild effects were found across European breeds (Riggio et al., 2014; Sallé et al., 2012, 2014) or in sheep lines bred for divergent susceptibility towards GIN infection (McRae et al., 2014). This genetic network likely causes functional trade‐offs between immune response and fitness as a result of pleiotropy, although weak positive (Assenza et al., 2014; Bishop et al., 1996; Bouix et al., 1998) or negative (Douch et al., 1995; Eady et al., 1998) genetic correlations between resistance and growth traits were found in domestic populations.

Environmental perturbations allow different genotypes to express maximal fitness across conditions, as a result from host genotype × environment (*G_h_* × *E*) interactions (Hoffmann & Merila, 1999; Lazzaro & Little, 2009; Lynch & Walsh, 1998; Seppälä & Jokela, 2016) or by inflecting the strength and direction of selection (Hayward et al., 2018). For instance, analyses of faecal egg count (FEC) data from commercial Merino sheep revealed increased heritability under the lowest and the highest parasite exposure, as a result from variation in sire estimated breeding values across environments (Hollema et al., 2018; Pollott & Greeff, 2004). In addition, exposure to unpredictable aversive events such as transportation, isolation or restraints is a common feature across farming systems (Proudfoot & Habing, 2015). This is known to induce chronic stress in sheep and significantly impact their behaviour (Destrez et al., 2013; Proudfoot & Habing, 2015). Because of the close links between chronic stress and the immune response in sheep (Ciliberti et al., 2017), unfavourable genetic correlations between the two traits may exist as found in birds (Buitenhuis et al., 2004). This could impact on the expression of resistance potential across farming conditions.

Third, selection for more resistant hosts could promote the rewiring of GIN species assemblage by reducing resistance to other parasite species (*G_h_* × *G_p_* interactions). Limited evidence from domestic Merino (Woolaston et al., 1990) or Romane sheep breeds (Gruner et al., 2004), however, suggest that selection for resistance to *H. contortus* confers significant but incomplete cross‐resistance to *Trichostrongylus colubriformis*, an intestinal GIN.

Therefore, abrupt variation in environmental conditions could release host cryptic variation overlooked under controlled conditions, and directional selection for host resistance could disrupt host‐parasite interactions. This is critical to the sustainability of the breeding option for the control of GIN populations. To investigate that matter, we created divergent sheep lines selected for resistance or susceptibility to *H. contortus* infection using three sires in each case. We monitored their resistance potential under chronic stress or against *T. colubriformis* infection following one and two rounds of divergence respectively. This was to model farming systems that would either differ in animal management (e.g. social isolation or animal restraint) or in the relative abundances of GIN species. Observations made on these lines suggest that sheep genetic potential was almost fully expressed under chronic stress, but significant *G_h_* × *G_p_* interactions occur.

Breeding for resistance is sustainable but allowance is to be made for environmental changes or worm species while breeding for resistance.

## 2 MATERIALS AND METHODS

### 2.1 Creation of two divergent lines of sheep

A full description of the selection scheme has been provided in the Appendix S1. Briefly, a QTL detection scan across European breeds identified eight regions (Table 1) significantly associated with resistance to GIN (Riggio et al., 2014). SNP from the 800K SNP chip located within these QTL regions (approximately 110 SNPs per region) were subsequently selected and genotyped using the KASP™ assay (LGC Genomics Ltd, UK) that consists in a competitive allele‐specific PCR (He et al., 2014). A nucleus Romane flock (ancestral generation G0, *n* = 274) was genotyped for these markers and phenotyped for resistance to *H. contortus* by two consecutive challenges with 10,000 larvae, as previously outlined (Sallé et al., 2012).

**TABLE 1:** Metadata associated with considered QTL for selection of G1 lambs

OAR	Region size	SNP count	Population	Trait—Original h^2^
3	4,993,965	106 (47,113)	SBF	Strongyle FEC ‐ 0.06
4	67,333,487	106 (635,222)	MBR, SBF, SDL	Strongyle FEC ‐ 0.02
5	5,928,484	110 (53,895)	MBR	*H. contortus* FECa ‐ 0.03
7	5,004,258	115 (43,515)	MBR	*H. contortus* FECa ‐ 0.03
12	12,858,699	250 (51,435)	MBR	*H. contortus* FECa ‐ 0.04
13	3,897,157	96 (40,595)	MBR	*H. contortus* FEC2 ‐ 0.05
14	5,128,189	105 (48,840)	SBF	*Nematodirus* sp FECa ‐ 0.07
21	2,919,789	10 (291,979)	MBR, SBF	Strongyle FEC ‐ 0.02 (MBR), 0.07 (SBF)

Note

QTL genomic (region size in bp, number of SNPs within region and average inter‐SNP distance in bp in brackets) and genetic metadata are listed for 8 regions considered to guide the selection of G_0_ individuals. Populations used for QTL detection are also listed (MBR, Martinik × Romane back‐cross; SBF, Scottish Blackface; SDL, Sarde × Lacaune back‐cross) with original trait and h^2^ estimates provided. FECa, faecal egg count (FEC) animal solution; FEC2, FEC at reinfection.

A marker‐assisted selection approach was subsequently applied to retain the most resistant and most susceptible G0 sires and ewes for conditional mating using single‐step GBLUP (Aguilar et al., 2010; Christensen & Lund, 2010). Instead of relying only on the genomic relationship matrix (VanRaden, 2008; Yang et al., 2010), this approach models the phenotype as the sum of fixed effects and a random animal effect estimated from a blended relationship matrix ***H*** that accounts for differences between pedigree and genomic information (Aguilar et al., 2010; Christensen & Lund, 2010). The relative weight given to the pedigree or genomic information is defined by a scaling parameter that ranges from 0 (pedigree only relationship matrix) to 1 (marker only relationship matrix) and was, in our case, set to 0.5 (Aguilar et al., 2010). The genomic relationship matrix used here consisted of the raw genomic information scaled by the parameter $k\;=\;2\sum_{i}^{n}p_{i}\left(1‐\;p_{i}\right)$, where $p_{i}$ refers to SNP *i* allele frequency. Matrix was then weighed to facilitate its inversion (VanRaden, 2008). As a summary, measured sheep phenotypes were hence modelled as the equal contribution of the selected 8 QTL and a polygenic effect accounting for the rest of the genome (estimated from pedigree information). Genomic estimated breeding values (***geBVs***) were computed for FEC at first and second infection and averaged to retain individuals with most extreme potentials.

Using these *geBVs*, the six most extreme G0 sires (three at each end of the geBV distribution) were mated with 55 and 63 resistant and susceptible G0 ewes respectively (among 118 females with breeding value estimations), to create 236 lambs (generation G1).

**FIGURE 1:**
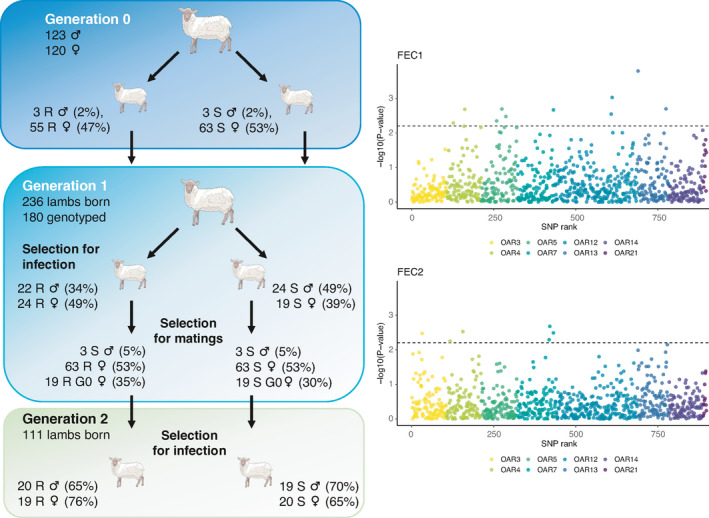
Overview of the selection process guided by 8 QTL. Left panels summarize the selection strategy to create divergent lines. At each selection step (to retain individuals enrolled in subsequent experimental infections or for matings), the number of sheep is given with corresponding selection intensities in brackets. The right panels show the association between 898 SNP within 8 QTL regions (materialized by colours) and faecal egg count at 1st (FEC1) and 2nd infection (FEC2) by *H. contortus* in G0 individuals. The dashed line corresponds to the retained statistical cut‐off for association. Sheep pictures were downloaded from https://smart.servier.com and used without any changes. Servier Medical Art by Servier is licensed under a Creative Commons Attribution 3.0 Unported License

Out of these 236 G1 lambs, a subset of 180 lambs were selected for genotyping with the 1000‐SNP chip, according to their expected breeding value (average of their parent breeding values, ***eBV***). eBVs for first and second infection FEC were computed using a model including known fixed effects (litter size, sex) and an individual random effect estimated from the pedigree relationship matrix. Their geBVs were subsequently estimated using their genotype information and the SNP effect calculated in G0. 87 G1 lambs were retained for the experiment based on their geBVs (Figure 1).

A second generation of lambs was produced following the mating of six G1 rams (three within each line, selected on their breeding values, **eBVs**) with 82 ewes selected among G1 ewes (*n* = 23 and 19 selected on their eBVs for both R and S lines respectively) and G0 ewes (*n* = 19 and *n* = 20 selected on their eBVs for both R and S lines respectively). A total of 111 lambs were created in generation G2 (from 55 ewes), out of which 80 lambs were subsequently selected within each line according to their expected eBVs (average of their parent breeding values). Analyses were run with AIREMLF90 and BLUPF90 for eBVs and geBVs estimations respectively (Misztal et al., 2002).

### 2.2 Behavioural treatment to establish how sheep resistance potential holds in stressful environments

Animal experiments and experimental procedures were approved by the French Ministry for Higher Education and Research and the Centre Val de Loire ethics committee under agreement numbers 2015010811379451_v4 and APAFIS#8973‐2017022108587640_v3. Behavioural treatment was applied to 84 enrolled G1 lambs allocated to four indoor pens, each housing 22 females or 20 males with equal proportion of lambs from both lines. Half of the sheep were submitted to a stress treatment or a control treatment 7 weeks prior to the first *H. contortus* infection. This behavioural treatment lasted throughout the experimental infection. The control treatment involved mild enrichment: sheep had access to a wool brush and were exposed to daily positive tactile contacts with humans. Twice a week, a familiar sheep keeper entered the pen, stayed passive and gave positive contacts to sheep that initiated contacts. The chronic stress treatment consisted in moving animals from their free‐range pens to individual cages where they remained locked down and isolated from their mates once a week. Isolation was applied until the end of the second artificial challenge and lasted 20 min in the first week, 40 min the following month and 10 min afterwards. This chronic stress treatment significantly impacted on sheep behaviour as measured by behavioural reactivity and standing‐lying behaviour (Appendix S2). Plasmatic cortisol level was not considered because of inconsistencies in its variation under chronic stress exposure.

### 2.3 Artificial infection with *Haemonchus contortus* and *Trichostrongylus colubriformis*

Lambs were kept indoors during the whole experiments to prevent natural GIN infection. The G1 lambs were challenged twice with 10,000 *H. contortus* infective larvae, given orally after 3 months of age. At the end of the first infection (30 days postinfection, dpi), lambs were drenched with ivermectin (2.5 ml/10 kg body weight of Oramec^®^, Merial, France) and left for a resting period of 2 weeks before another infection took place with the same infection dose. Lambs were weighed on the same day and Faecal Egg Counts were quantified at 24 and 30 dpi.

To evaluate whether genetic resistance was sustained in the face of an intestinal GIN, the second generation of sheep from both divergent lines were either infected by *H. contortus*, or by 10,000 infective *T. colubriformis* larvae. In the latter case, FEC were measured at 24, 30 dpi after the first infection and at 30 dpi after the second infection. Lambs were weighed before and at 14 and 30 days postinfection.

### 2.4 Statistical analyses

Statistical analyses were implemented with R v3.5 unless stated otherwise (R Core Team, 2016). For every analysis, raw FEC data were normalized by a fourth‐root transformation that outperformed the logarithmic transformation (Shapiro–Wilk's test ranging between 0.96 and 0.90 for fourth‐root transformed FEC but 0.90 and 0.84 for log‐transformed FEC). Summary statistics of considered variables and detailed modelling outputs have been provided in Tables 1, 2 and Table S2 respectively. Considered response variables were either average FEC at first or second infection (average between measures taken at 24 and 30 days postinfection) or across the two infections (average across infections).

**TABLE 2:** Measured raw and 4th root transformed FEC in G_0_ and divergent lines

	Generation	Line	Mean	Std. deviation	Minimum	Maximum
Raw FEC	G0		9569	5956	50	32900
	G1	R	424	594	0	2264
	G2	R	1000	1196	0	3900
	G1	S	3177	2213	199	8272
	G2	S	3999	1897	937	7750
4th‐root FEC	G0		9.44	1.82	2.66	13.47
	G1	R	3.60	1.76	0.00	6.90
	G2	R	4.49	2.32	0.00	7.90
	G1	S	7.03	1.65	3.76	9.54
	G2	S	7.79	0.98	5.53	9.38

Note

FEC, faecal egg count; G0, nucleus flock; G1 and G2 correspond to first and second round of divergent selection; R and S stand for resistant and susceptible respectively.

### 2.5 Association study in the G_0_ population

A regression‐based association study was performed between FEC measured at first and second infection in the G_0_ population to evaluate the segregation of the targeted QTL and their effect in the G_0_ population. At each polymorphic marker (*n* = 898, Table S1), phenotypic data were regressed upon fixed effects (sex, feeding lot and lamb suckling modality, i.e. artificial or not), a polygenic effect estimated from all SNPs (accounting for population structure) and the marker effect as already implemented elsewhere (Legarra et al., 2015). SNP substitution estimates and associated standard errors were used to derive P‐values from a Student's *t*‐test statistic. QTL heritability was estimated by aggregating the ratios of additive genetic variance to phenotypic variance estimated as part of the REML procedure run for each SNP. For the sake of comparison between FEC at first and second infection, we considered a cut‐off corresponding to a Bonferroni correction for 8 independent tests (*p*‐value = 6.25 × 10^−3^).

### 2.6 Response to selection

To estimate phenotypic divergence in FEC between sheep lines, individual *H. contortus* infection data were pre‐corrected for fixed environmental effects, *that is* sex, milking mode (artificial or not) and generation (accounting for year effect), and an individual random effect, using the nlme package v3.1‐140 (Pinheiro et al., 2019). Individual random effects were standardized to unselected G0 mean and standard deviation.

Responses to selection for FEC at first and second infection or across infections were evaluated within each generation and each line by regressing individual random effects from *H. contortus*‐infected offspring upon their respective midparent values, computed as the average value of each lamb's sire and dam (Falconer & Mackay, 1996). This regression coefficient provides an estimate for realized heritability (Falconer & Mackay, 1996) and was used to establish the asymmetry of response between resistant and susceptible lines.

To estimate the expected genetic gain across infection, we computed the mean genetic gain between first and second infection. We considered pedigree‐based breeding values (eBV) as they were available across generations (geBVs were only available for G0 and G1 individuals) and were strongly correlated with geBV. eBVs were estimated from recorded phenotypes in G0, G1 and G2 individuals using a mixed model including fixed environmental effects and a random individual effect estimated from the pedigree relationship matrix (encompassing 1559 individuals) as implemented in the AIReml software (http://nce.ads.uga.edu/wiki/doku.php?id=readme.aireml).

Genetic gain within each line (*R_line_*) was expressed in genetic standard deviation (σ_g_) and was estimated as the cross‐product between the overall selection intensity *i* (average of selection intensities in males, *i_m_*, and in females, *i_f_*) and the selection accuracy *h* (Falconer & Mackay, 1996) as:

$R_{\mathrm{line}}\;=\;{0.5\times [h}_{FEC1}\times 0.5\times \left(i_{f}\;+\;i_{m}\right)]\;+{0.5\times [h}_{FEC2}\times 0.5\times \;\left(i_{f}\;+\;i_{m}\right)]$, with *h_FEC1_* and *h_FEC2_* corresponding to the square‐root of estimated heritabilities for FEC measured at first infection ($h_{FEC1}^{2}$= 0.52 ± 0.11) and second infection ($h_{FEC2}^{2}$= 0.33 ± 0.07).

To create the G_1_ population, 2% of the G_0_ males (*i_m_* = 2.421) were mated to 47% (*i_f_* = 0.846) and 53% (*i_f_* = 0.75) of the resistant and susceptible G_0_ females respectively (Figure 1, Appendix S1). The creation of G2 individuals involved 5% of the G_1_ males (*i_m_* = 2.063). These were mated with 19 G_0_ (*i_f_* = 1.059 and 1.259 for the resistant and susceptible lines respectively) and 63 G_1_ females in both lines (Figure 1, Appendix S1). To account for this, the expected genetic gain was computed as the sum of the genetic gains from each type of mating (G_1_ × G_1_ or G_1_ × G_0_) following the aforementioned equation, weighing each *i_f_* coefficient by the respective proportion of females, *that is* 23% and 72% for G_0_ and G_1_ females.

For both G1 and G2 generations, total expected divergence was derived as the sum of expected responses from resistant and susceptible lines.

To estimate putative trade‐offs between resistance to GIN and lamb weight, measured body weights were modelled using a mixed model with repeated measures, including fixed effects (litter size, sex, generation, day postinfection, and an effect aggregating line and corresponding generation), and a random effect accounting for interindividual variations. To account for differences in body weight at the beginning of the trial, weight data measured before any infection took place was fitted as a covariate.

### 2.7 Sheep line × Environment (*G_h_* × *E*) and sheep line × Parasite (*G_h_* × *G_p_*) interactions

To test for *G_h_* × *E* and *G_h_* × *G_p_* interactions, normalized FEC data collected at every time point (24‐ or 30‐day postinfection) were scaled within each experimental block (infection rank, day postinfection and considered environment) to prevent spurious signals from heterogeneous variances between blocks (Pollott & Greeff, 2004). A mixed model for repeated measures was built, whereby scaled normalized FEC were regressed upon two interaction terms (between the lamb genetic line and the time postinfection, and between the lamb genetic line and the environment), and an additional random effect accounting for inter‐individual variation.

Behaviour data were considered as normally distributed (Shapiro‐Wilk's test ranging between 0.91 and 0.98). Physical contact records were however skewed towards 0 and were thus binary encoded, *that is* 0 or 1 in absence or presence of contact with their mates or with the operator, and modelled using a logistic regression framework. To test for the effect of behaviour treatment, recorded behaviour data were regressed upon sheep line, sex and day and their interactions, and a random effect accounting for inter‐individual variation. Regression models were built following stepwise variable selection procedure that aims to find the model with minimal Akaike Information Criterion (AIC). Bleating records in phase 1 of the test were also corrected for their initial value before chronic stress treatment took place to correct for the increased bleating in susceptible lambs from the stress group.

Pearson's correlations were estimated with the *rcorr()* function from the Hmisc package (Harrell & Dupont, 2017).

## 3 RESULTS

### 3.1 Overview of the selection process

An overview of the selection process is showcased in Figure 1 and the detailed breakdown of individuals retained for G1 and G2 creation can be found in the Appendix S1. The most striking features of this procedure are recalled herein. The G0 nucleus flock was genotyped for 8 QTL regions (Table 1) covered with approximately 100 SNPs each. SNPs were positioned between 40,595 and 53,895 bp apart for most regions but on OAR4 and OAR21 (mean distance of 635,222 and 291,979 bp respectively). Estimated QTL heritabilities ranged between 0.02 and 0.07 in the populations they were originally found to be segregating in (Table 1). An association study performed in G0 individuals found statistical associations (*p* < 6.3 × 10^−3^) between FEC at first infection and most QTL (OAR4, 5, 7, 12 and 13; genomic inflation factor = 1.19; Figure 1, Figure S1). The only QTL found on OAR3, 4 and 7 reached the same threshold for FEC at second infection (genomic inflation factor = 1.14, Figure 1, Figure S1) with lower associated explained variances (Table 1). From these estimates, the overall QTL heritability was 0.14 ± 0.04 and 0.02 ± 0.02 for FEC at first and second infection respectively. This was lower than that found using both molecular and pedigree information in that population (*h* ^2^ = 0.52 ± 0.11 and 0.33 ± 0.07 for the two traits respectively).

### 3.2 Achieved divergence and response to selection

**FIGURE 2:**
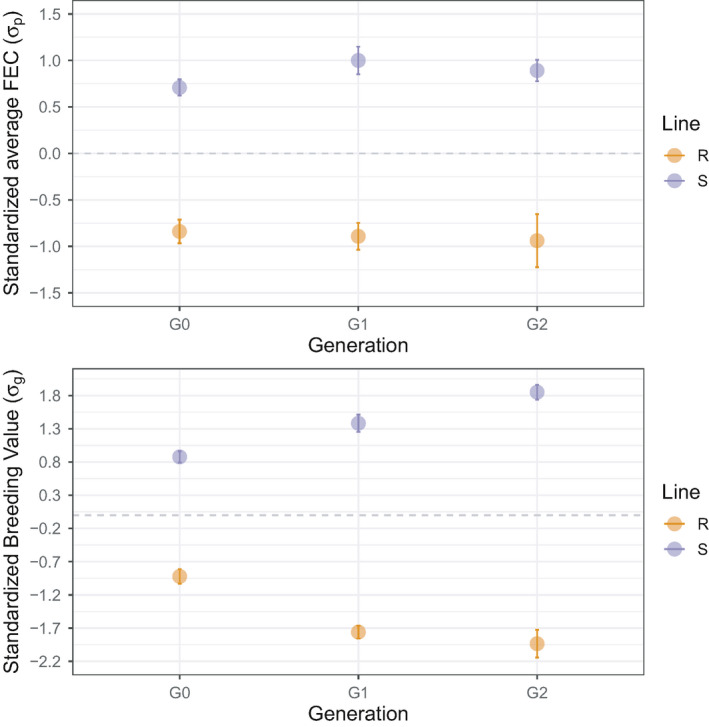
Achieved phenotypic and genetic divergences between sheep lines across generations. Top panels show the corrected average faecal egg counts (mean ± SE) observed across two infections for parental (G0), first (G1) and second (G2) generations. Bottom panels represent the distribution of estimated breeding values (eBVs) using the pedigree information only. For the sake of comparison, FEC and eBVs data were scaled (mean centred and reduced to G0 standard deviation unit). Grey dashed line indicates G0 mean value

To evaluate the response to selection in both lines and our selection procedure accuracy, we compared the performance of both R and S sheep infected with *H. contortus* (Figure 2, Figure S2). G1 and G2 generations significantly diverged from the unselected G0 nucleus on the phenotypic scale: R lambs of respective generations showed FEC reduction of 0.62 σ_p_ (*t*_70_ = −3.8, *p* = 2 × 10^−3^) and 0.67 σ_p_ (*t*_20_ = −2.8, *p* = 5 × 10^−3^) relative to the G0 nucleus flock, and S lambs FEC increased by 0.72 σ_p_ (*t*_55_ = 4.99, *p* = 10^−6^) and 0.61 σ_p_ (*t*_31_ = 3.11 *p* = 2 × 10^−3^) relative to their G0 unselected relatives (Figure 2). This corresponded to phenotypic divergence between R and S lamb FEC across infection of 1.89 and 1.87 σ_p_ for G1 and G2 generations respectively (Figure 2). Accordingly, 3.14 and 3.8 genetic standard deviations (σ_g_) were found between R and S lines at G1 and G2 generations (Figure 2). This was slightly higher than the expected respective genetic gains of 2.08 and 2.87 σ_g_ for G1 and G2 populations. This difference mirrors the truncation sampling performed within each population to create the experimental groups.

Despite similar selection intensity in R and S lines, observed response to selection for average FEC across infection was asymmetrical between lines in G2 lambs (Figure 2). This was accompanied by a reduced variance in FEC upon reinfection (Figure S2), with measured values at 30 dpi remaining high in a range between 1,650 and 14,250 eggs/g (median FEC = 5,625 eggs/g). On the contrary, the resistant line achieved half the response of their susceptible counterpart for FEC across infections (0.38 σ_p_ ± 0.27 and 0.82 σ_p_ ± 0.27 for the resistant and susceptible lines, respectively, Table S2). This was the result of more variable FEC in that line (Figure 2, Figure S2) that was also evident at first and second infection (Figure S2, Table S2). This lower response could not be related to inbreeding that was not significantly different between G2 R and S lines (*t*_35_ = −2.01, *p* = 0.05).

Of note, estimated geBVs and eBVs showed strong correlation (*r*_241_ = 91% and 71% for FEC at first and second infection, respectively, *p* < 10^−4^). The lower correlation for FEC at reinfection corroborated the lower overall association between SNPs and this trait in the G0 population (Figure 1). This reduced association between SNPs and FEC at 2nd infection may contribute to the lesser divergence found for that trait between R and S individuals in G1 lines (Figure S1).

### 3.3 Trade‐off between resistance to *Haemonchus contortus* and lamb growth

**FIGURE 3:**
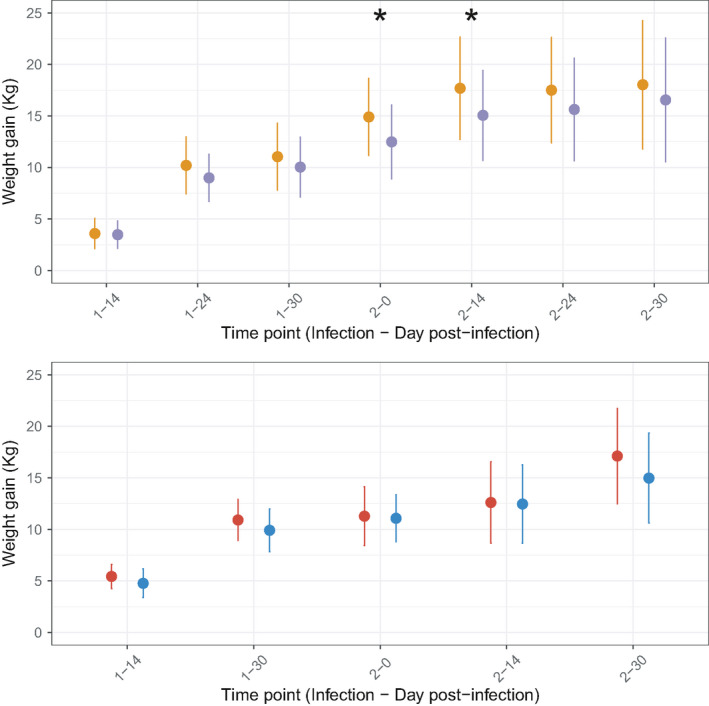
Weight gain trajectories measured in resistant and susceptible lambs following *Haemonchus contortus* infection. Weight gain (mean ± SE) from day 0 are presented for resistant (orange or red) and susceptible (purple or blue) sheep lines. Phenotypes recorded on first‐generation lambs (G1; *n* = 23 and 20 individuals for resistant and susceptible groups respectively) are given in top panels, and bottom panels present that measured on second‐generation lambs (G2; *n* = 18 and 20 individuals for resistant and susceptible groups respectively). Asterisks indicate significant reduction in the susceptible individuals

Lambs were weighted to establish putative trade‐offs between FEC and lamb growth (Figure 3). Analysis of their weight trajectories showed that they were not statistically different between selected lines (average weight differences of 568 g, *t*_38_ = −0.61, *p* = 0.54 in G1 and of 1.27 kg, *t*_34_ = −1.31, *p* = 0.19 for G2 lambs), suggesting that higher resistance was not detrimental to production traits (Figure 3, Table S2). On the contrary, weight gains were reduced in susceptible G1 individuals (gain difference ranging between 2.3 and 2.5 kg, *p* < 0.05; Table S2) in the early phase of the second infection (Figure 3).

However, the G2 R lambs were significantly lighter than their susceptible counterparts before any infection took place (4.3 kg difference, *F*_1,35_ = 8.99, *p* = 5 × 10^−3^). This was not related to age difference between both groups (136 days on average in R and S groups, *t*_73_ = 0, *p* = 0.99). It hence appears that selection for resistance did not impact on sheep growth curve although lighter lambs were obtained in the R line after two rounds of divergence.

### 3.4 Limited effect of chronic stress on sheep resistance but significant interaction following *Trichostrongylus colubriformis* infection

To identify putative G × E interactions, related individuals with divergent resistance to *H. contortus* infection were exposed to various environments, *that is* chronic stress or the intestinal parasite *T. colubriformis*. Half of the G1 selected lambs were exposed to chronic and unexpected stresses, while the other half were maintained under controlled environmental conditions.

**FIGURE 4:**
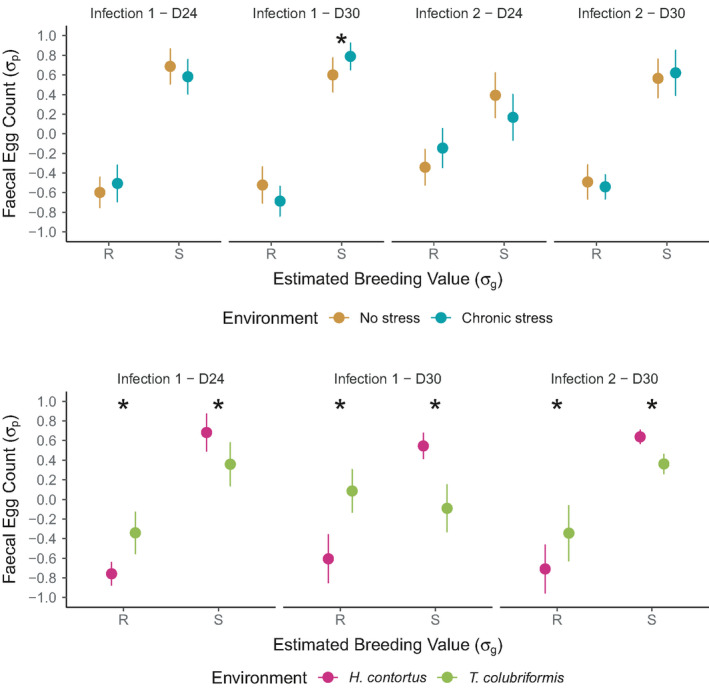
Genetic divergence variation across environments. Average faecal egg counts (FEC, mean ± SE) are plotted across considered time points (infection rank‐day postinfection) and environments for resistant (circles) and susceptible (triangles) sheep lines. Top panels correspond to the effect of exposure to chronic stress and bottom panels illustrate the impact of infection by another parasite species. Raw FEC data were normalized with a 4th root transformation and scaled (mean centred and reduced to standard deviation unit) within each group × time point. eBVs were scaled within each trial. Asterisks indicate significant group × environment interaction term

Behaviour modifications are described in full under the Appendix S2 with statistical details listed in Table S2. Despite significant alterations in sheep behaviour following chronic stress exposure, limited interactions were found between genetic line and their environment (Figure 4; Figure S3). Indeed, the relationship between genetic line and transformed FEC did not significantly differ across conditions (*F*_1,82_ = 0.03, *p* = 0.87). However, phenotypic divergence between lines exposed to chronic stress significantly decreased 24 days after the second infection (Figure 4). In that case, susceptible lambs excreted less eggs (−0.66 σ_p_, *t*_252_ = −2.91, *p* = 4 × 10^−3^) than their sibs maintained under controlled conditions (Figure 4). This would be compatible with limited genetic correlations between response to chronic stress exposure and FEC. Of note, the magnitude of *G_h_* × *E* interactions were statistically different between sire families (Figure S4): progenies of two susceptible sires displayed higher phenotypic variability following exposure to stressful conditions (Figure S4). In some sire families chronic stress appeared to be beneficial (interaction term equal to −1.16 σ_p_, *p* = 0.024 for sire S‐132550) while in others was detrimental (interaction term equal to 0.65 σ_p_, *p* = 0.046 for sire S‐132361; Figure S4).

The second trial aimed to investigate whether the genetic potential for resistance or susceptibility to *H. contortus* infection would be sustained in the face of another GIN species (Figure 4). Of note, *T. colubriformis* infection yielded fewer eggs (average FEC of 411 eggs/g across conditions, ranging between 0 and 1,100 eggs/g) as a result of the lower fecundity of this parasite species. The phenotypic divergence between R and S sheep remained significant across considered GIN species (1.52σ_p_ difference, *F*_1,73_ = 34.9, *p* = 3 × 10^−8^) but was largely driven by the existing divergence between *H. contortus* infected individuals. Indeed, the phenotypic expression of lamb genetic potential was significantly reduced after infection by the intestinal *T. colubriformis* species. In that case, lambs genetically susceptible to *H. contortus* were less affected by *T. colubriformis* infection than their susceptible counterparts, as evidenced by the significant *G_h_* × *G_p_* effect (−0.9 σ_p_, *F*_1,73_ = 9.77, *p* = 0.003; Figure 4). Overall, mild difference in egg counts were measured between both lines (138 eggs/g difference, *t*_36_ = 1.92, *p* = 0.06) with significant differences observed at 24 days postinfection at first infection (*t*_36_ = −2.30, *p* = 0.027) and 30 days postinfection upon reinfection (*t*_36_ = −2.38, *p* = 0.026).

On the other hand, we found no evidence that the magnitude of *G_h_* × *G_p_* interactions varied across families.

These two trials hence provide evidence that the phenotypic expression of *H. contortus*‐resistant individuals holds in the face of chronic stress, but can vary across families. It also offers a significant advantage towards infection by a different intestinal GIN species although to a lower extent than for the species used during selective breeding.

## 4 DISCUSSION

Understanding how the host–parasite system behaves following changes in their respective environments is central to ensure sustainable control of GIN in livestock through animal breeding. Our work investigated how directional selection for contrasting levels of resistance to GIN infection would affect expression of sheep potential towards environmental change.

Our design relied on divergent sheep lines that provide a model system to quickly evaluate consequences of such environmental variation. Following two rounds of selection, we observed significant divergence on both phenotypic and genetic scales. We obtained aggregated estimates of the genetic variance across environments, either quantified by the interaction between lamb genetic groups and their environment, or by sire reaction norms across environments. This represents a first glimpse into the contribution of directional selection to genetic × environment interactions. Because of the infancy of these lines and the need to apply high selection intensity, observations were made on a limited number of generations and a few sire families. Despite the lack of replication and the contribution of drift to our design, our findings were in good agreement with past independent observations. In addition, pushing the divergence selection process further in this flock to increase divergence may be limited by the contribution of inbreeding depression to parasite resistance (Coltman et al., 1999). Our trial did not evidence major effects of chronic stress on sheep resistance, but we found significant interactions between exposures to diverse parasite species. Trade‐offs were limited although resistant lambs exhibited reduced body weight after the second round of selection before infection took place. This would corroborate previous observations from divergent selection lines in the Romney breed that indicated reduced growth rate in resistant individuals (Bisset et al., 2001). A definitive conclusion on the impact of selection for enhanced resistance on growth traits is difficult as other genetic correlations estimated under natural infection conditions with different breeds were either positive or nonsignificant (Bishop et al., 1996; Bouix et al., 1998; Pollott et al., 2004; Robinson et al., 2009).

Of note, the considered QTL were mostly associated with FEC at first infection in the founder population while their heritability for FEC at 2nd infection was negligible. Because G1 selection was partly driven by these regions, it would corroborate the higher divergence observed for that trait despite accounting for the polygenic effect (through the simultaneous use of pedigree information) in our single‐step selection procedure. Altogether, these QTL would account for a quarter of available additive genetic variance for FEC in naive animals challenged with *H. contortus*.

The response to selection was asymmetrical in G2 lambs. It yielded an increased divergence towards susceptibility rather than resistance, despite similar selection intensity across sexes and lines within generations. In the absence of replicated lines, it remains difficult to disentangle this observation from the differential contribution of random drift within each line (Falconer & Mackay, 1996). This may also indicate that the proportion of phenotypic variance of genetic origin is more difficult to estimate for resistant individuals than for susceptible lambs. Indeed, resistance is measured from FEC, whose distribution will be censored to 0 across a range of resistance levels, thereby hampering variance estimation. It is also possible that the selection applied to the pleiotropic gene networks underpinning the immune response to GIN infection (Lazzaro & Little, 2009; Sallé et al., 2012; Sparks et al., 2019) could yield asymmetric phenotypic expression upon selection. Additional rounds of divergence would be needed to support this hypothesis. Another aspect that could contribute to this pattern might come from maternal effects, *for example* carry‐over effect linked to the transfer of antibodies through the milk (Sparks et al., 2020). However, estimation of this effect was beyond the scope of our study and would require many more observations by ewes, that is temporal replicates. Beside, maternal effect represents a limited fraction of FEC genetic variance (Ciappesoni et al., 2013; Ngere et al., 2018) and it vanishes after the lamb reaches 3 months of age (Bishop et al., 1996) as in our design. Characterizing milk composition and antibody transfer in ewes from both lines would contribute to a deeper understanding of these effects.

In line with previous reports (Gruner et al., 2004; Woolaston et al., 1990), susceptibility levels towards *H. contortus* infection were well correlated to that measured upon *T. colubriformis* infection and we found no indication of between sire variations across environments. Yet, lambs selected for diverging susceptibility to *H. contortus* did not express the same divergence towards *T. colubriformis* infection. Previous estimated genetic correlations between FEC of both GIN species were positive but ranged between 0.9 and 0.76 at first and second infection (Gruner et al., 2004). This indicates that a large common genetic background is associated with resistance to both GIN species, but that a minor species‐specific genetic component contributes to immune mechanisms associated with either the GIN species or with its niche (abomasum in one case or the small intestine in the other) or both. In contrast, only 38 genes were found differentially expressed across two divergent sheep lines bred for resistance to *H. contortus* or *T. colubriformis* and infected with *H. contortus* upon primary infection (Zhang et al., 2019). This intersecting set vanished following reinfection as differentially expressed genes were private to each sheep line (Zhang et al., 2019). These lines of evidence would suggest that despite a largely common genetic architecture between resistances to both GIN species, selection for resistance to one species may result in an efficient nonadaptive response at the transcriptomic level upon abrupt environmental modification (Ghalambor et al., 2007). In our experiment, observed plasticity would also result from a similar maladaptive response originating from partially correlated traits in lambs selected for resistance to one species and exposed to another. This nonadaptive response may highlight cryptic mechanisms that have been maintained through time by selection, to provide selective advantage against seasonal variation in GIN community structures (O’Connor et al., 2006). Selection would hence have acted on plasticity itself (Lynch & Walsh, 1998).

Of note, we did not evidence systematic difference in sheep phenotypes following chronic stress exposure but a reduced susceptibility of stressed lambs upon reinfection and within‐family variation in their differential FEC response upon isolation. This lack of major interaction may reflect the moderate stress response induced by the applied behavioural treatment. However, social isolation is known to induce strong stress response (Minton & Blecha, 1990) or pessimistic judgement bias (Doyle et al., 2010) in sheep and similar experimental setting as ours previously induced strong chronic stress response (Guesdon et al., 2015). In addition, the stress response induced by social isolation depends on an individual's ability to develop a coping strategy. Variation of this ability could have obscured putative interactions between the behavioural treatment and measured FEC. The mild interactions found (at the family level) may also reflect the limited effect of selection after a single generation of divergence. Because we aimed to maximize the selection intensity (thereby reducing the number of lambs available) and because of technical limitations (behavioural treatment and phenotyping applied on more than 80 lambs), it was not possible to estimate genotype x environment interactions at every generation. Nonetheless, the estimated departure between genotypes across environments was limited. As such it would be compatible with tight genetic correlation between FEC measured with or without exposure to chronic stress that are unlikely to be detected after a second round of divergence. The complex interactions between the immune response and chronic stress is primarily driven by the hypothalamic‐pituitary‐adrenal and the sympathetic–adrenal medullary axes that, respectively, control the release of glucocorticoids and catecholamines (Khansari et al., 1990; Padgett & Glaser, 2003). The intricacies of both neuronal and immune system have not been fully resolved, but evidence suggests that the glucocorticoid corticosterone dampens the immune response by promoting a shift from a pro‐inflammatory Th‐1/Th‐17 response to a Th‐2 response (Elenkov, 2004; Harpaz et al., 2013; Padgett & Glaser, 2003). This latter polarization is associated with beneficial outcome of GIN infection and enhanced in more resistant hosts, as reported in mice (Filbey et al., 2014) or in sheep (Terefe et al., 2007). The decrease in FEC observed in lambs exposed to chronic stress 24 days after reinfection is compatible with a delayed worm development that could be underpinned by an enhanced Th‐2 response in these individuals. However, the lack of any beneficial effect at primary infection and the transient nature of this observation upon reinfection warrant further validation. Because of the variations in the extent and polarization of glucocorticoid level changes following chronic stress (Doyle et al., 2011; Terlouw et al., 1991), such validation would require technical developments including cortisol dosage from the wool as proposed for humans (Russell et al., 2012).

Our experimental trial found limited genetic x environment interactions but significant host x parasite interactions. This suggests that the selection of more resistant sheep would be relatively insensitive to farming management practices, although a few individuals may have an increased environmental sensitivity. On the contrary, the composition of GIN communities could limit the benefits of the selection strategy. Additional environmental disruption like feeding restriction could be investigated to confirm that the resistant potential holds under resource‐limited environments.

## CONFLICT OF INTEREST

The authors declare no conflicts of interest.

## Supporting information

Supplementary Material

Table S1

Table S2

## Data Availability

R scripts used for raw data analysis and associated data files are freely available at https://github.com/guiSalle/GEMANEMA.

## References

[eva13294-bib-0001] Aguilar, I. , Misztal, I. , Johnson, D. L. , Legarra, A. , Tsuruta, S. , & Lawlor, T. J. (2010). Hot topic: A unified approach to utilize phenotypic, full pedigree, and genomic information for genetic evaluation of Holstein final score. Journal of Dairy Science, 93, 743–752. 20105546 10.3168/jds.2009-2730

[eva13294-bib-0002] Assenza, F. , Elsen, J. M. , Legarra, A. , Carre, C. , Salle, G. , Robert‐Granie, C. , & Moreno, C. R. (2014). Genetic parameters for growth and faecal worm egg count following *Haemonchus contortus* experimental infestations using pedigree and molecular information. Genetics Selection Evolution, 46, 13. 10.1186/1297-9686-46-13 PMC3939629 24528625

[eva13294-bib-0003] Bishop, S. C. (2012). A consideration of resistance and tolerance for ruminant nematode infections. Frontiers in Genetics, 3, 168. 10.3389/fgene.2012.00168 23248638 PMC3522420

[eva13294-bib-0004] Bishop, S. C. , Bairden, K. , McKellar, Q. A. , Park, M. , & Stear, M. J. (1996). Genetic parameters for faecal egg count following mixed, natural, predominantly *Ostertagia circumcincta* infection and relationships with live weight in young lambs. Animal Science, 63, 423–428.

[eva13294-bib-0005] Bisset, S. , Morris, C. , McEwan, J. , & Vlassof, A. (2001). Breeding sheep in New Zealand that are less reliant on anthelmintics to maintain health and productivity. New Zealand Veterinary Journal, 49, 236–246. 10.1080/00480169.2001.36238 16032198

[eva13294-bib-0006] Blake, N. , & Coles, G. (2007). Flock cull due to anthelmintic‐resistant nematodes. The Veterinary Record, 161, 36. 10.1136/vr.161.1.36-b 17617547

[eva13294-bib-0007] Bouix, J. , Krupinski, J. , Rzepecki, R. , Nowosad, B. , Skrzyzala, I. , Roborzynski, M. , Fudalewicz‐Niemczyk, W. , Skalska, M. , Malczewski, A. , & Gruner, L. (1998). Genetic resistance to gastrointestinal nematode parasites in Polish long‐wool sheep. International Journal for Parasitology, 28, 1797–1804. 10.1016/S0020-7519(98)00147-7 9846618

[eva13294-bib-0008] Buitenhuis, A. J. , Rodenburg, T. B. , Wissink, P. H. , Visscher, J. , Koene, P. , Bovenhuis, H. , Ducro, B. J. , & van der Poel, J. J. (2004). Genetic and phenotypic correlations between feather pecking behavior, stress response, immune response, and egg quality traits in laying hens. Poultry Science, 83, 1077–1082. 10.1093/ps/83.7.1077 15285495

[eva13294-bib-0009] Christensen, O. F. , & Lund, M. S. (2010). Genomic prediction when some animals are not genotyped. Genetics, Selection, Evolution: GSE, 42, 2. 10.1186/1297-9686-42-2 PMC2834608 20105297

[eva13294-bib-0010] Ciappesoni, G. , Goldberg, V. , & Gimeno, D. (2013). Estimates of genetic parameters for worm resistance, wool and growth traits in Merino sheep of Uruguay. Livestock Science, 157, 65–74. 10.1016/j.livsci.2013.07.011

[eva13294-bib-0011] Ciliberti, M. G. , Albenzio, M. , Inghese, C. , Santillo, A. , Marino, R. , Sevi, A. , & Caroprese, M. (2017). Peripheral blood mononuclear cell proliferation and cytokine production in sheep as affected by cortisol level and duration of stress. Journal of Dairy Science, 100, 750–756. 10.3168/jds.2016-11688 27865492

[eva13294-bib-0012] Coltman, D. W. , Pilkington, J. G. , Smith, J. A. , & Pemberton, J. M. (1999). Parasite‐mediated selection against inbred Soay sheep in a free‐living island populaton. Evolution; International Journal of Organic Evolution, 53, 1259–1267. 10.1111/j.1558-5646.1999.tb04538.x 28565537

[eva13294-bib-0013] Destrez, A. , Deiss, V. , Leterrier, C. , Boivin, X. , & Boissy, A. (2013). Long‐term exposure to unpredictable and uncontrollable aversive events alters fearfulness in sheep. Animal, 7, 476–484. 10.1017/S1751731112001796 23031226

[eva13294-bib-0014] Douch, P. G. , Green, R. S. , Morris, C. A. , & Hickey, S. M. (1995). Genetic factors affecting antibody responses to four species of nematode parasite in Romney ewe lambs. International Journal for Parasitology, 25, 823–828. 10.1016/0020-7519(94)00213-8 7558568

[eva13294-bib-0015] Doyle, R. E. , Lee, C. , Deiss, V. , Fisher, A. D. , Hinch, G. N. , & Boissy, A. (2011). Measuring judgement bias and emotional reactivity in sheep following long‐term exposure to unpredictable and aversive events. Physiology & Behavior, 102, 503–510. 10.1016/j.physbeh.2011.01.001 21236279

[eva13294-bib-0016] Doyle, R. E. , Vidal, S. , Hinch, G. N. , Fisher, A. D. , Boissy, A. , & Lee, C. (2010). The effect of repeated testing on judgement biases in sheep. Behavioural Processes, 83, 349–352. 10.1016/j.beproc.2010.01.019 20117188

[eva13294-bib-0017] Eady, S. J. , Woolaston, R. R. , Ponzoni, R. W. , Lewer, R. P. , Raadsma, H. W. , & Swan, A. A. (1998). Resistance to nematode parasites in Merino sheep: Correlation with production traits. Australian Journal of Agricultural Research, 49, 1201.

[eva13294-bib-0018] Elenkov, I. J. (2004). Glucocorticoids and the Th1/Th2 Balance. Annals of the New York Academy of Sciences, 1024, 138–146. 10.1196/annals.1321.010 15265778

[eva13294-bib-0019] Falconer, D. S. , & Mackay, T. F. C. (1996). Introduction to quantitative genetics (4th ed.). Pearson Education Limited.

[eva13294-bib-0020] Filbey, K. J. , Grainger, J. R. , Smith, K. A. , Boon, L. , Rooijen, N. , Harcus, Y. , Jenkins, S. , Hewitson, J. P. , & Maizels, R. M. (2014). Innate and adaptive type 2 immune cell responses in genetically controlled resistance to intestinal helminth infection. Immunology and Cell Biology, 92, 436–448. 10.1038/icb.2013.109 24492801 PMC4038150

[eva13294-bib-0021] Ghalambor, C. K. , McKay, J. K. , Carroll, S. P. , & Reznick, D. N. (2007). Adaptive versus non‐adaptive phenotypic plasticity and the potential for contemporary adaptation in new environments. Functional Ecology, 21, 394–407. 10.1111/j.1365-2435.2007.01283.x

[eva13294-bib-0022] Gold, S. , Regan, C. E. , McLoughlin, P. D. , Gilleard, J. S. , Wilson, A. J. , & Poissant, J. (2019). Quantitative genetics of gastrointestinal strongyle burden and associated body condition in feral horses. International Journal for Parasitology. Parasites and Wildlife, 9, 104–111. 31011533 10.1016/j.ijppaw.2019.03.010 PMC6462499

[eva13294-bib-0023] Gruner, L. , Bouix, J. , & Brunel, J. C. (2004). High genetic correlation between resistance to *Haemonchus contortus* and to *Trichostrongylus colubriformis* in INRA 401 sheep. Veterinary Parasitology, 119, 51–58. 10.1016/j.vetpar.2003.10.014 15036576

[eva13294-bib-0024] Guesdon, V. , Meurisse, M. , Chesneau, D. , Picard, S. , Lévy, F. , & Chaillou, E. (2015). Behavioral and endocrine evaluation of the stressfulness of single‐pen housing compared to group‐housing and social isolation conditions. Physiology & Behavior, 147, 63–70. 10.1016/j.physbeh.2015.04.013 25865708

[eva13294-bib-0025] Harpaz, I. , Abutbul, S. , Nemirovsky, A. , Gal, R. , Cohen, H. , & Monsonego, A. (2013). Chronic exposure to stress predisposes to higher autoimmune susceptibility in C57BL/6 mice: Glucocorticoids as a double‐edged sword: Immunomodulation. European Journal of Immunology, 43, 758–769. 23255172 10.1002/eji.201242613

[eva13294-bib-0026] Harrell, F. E. , & Dupont, C. (2017). Hmisc : Harrell Miscellaneous. Consulté à l’adresse Retrieved from https://github.com/harrelfe/Hmisc/

[eva13294-bib-0027] Hayward, A. D. , Pemberton, J. M. , Berenos, C. , Wilson, A. J. , Pilkington, J. G. , & Kruuk, L. E. B. (2018). Evidence for selection‐by‐environment but not genotype‐by‐environment interactions for fitness‐related traits in a wild mammal population. Genetics, 208, 349–364. 10.1534/genetics.117.300498 29127262 PMC5753868

[eva13294-bib-0028] He, C. , Holme, J. , & Anthony, J. (2014). SNP genotyping: The KASP assay. Methods in Molecular Biology (Clifton. N.J.), 1145, 75–86. 10.1007/978-1-4939-0446-4_7 24816661

[eva13294-bib-0029] Hoffmann, A. A. , & Merila, J. (1999). Heritable variation and evolution under favourable and unfavourable conditions. Trends in Ecology & Evolution, 14, 96–101. 10.1016/S0169-5347(99)01595-5 10322508

[eva13294-bib-0030] Hollema, B. L. , Bijma, P. , & van der Werf, J. H. J. (2018). Sensitivity of the breeding values for growth rate and worm egg count to environmental worm burden in Australian Merino sheep. Journal of Animal Breeding and Genetics = Zeitschrift Fur Tierzuchtung Und Zuchtungsbiologie, 135, 357–365. 10.1111/jbg.12349 29993145

[eva13294-bib-0031] Kaplan, R. M. , & Vidyashankar, A. N. (2012). An inconvenient truth: Global worming and anthelmintic resistance. Veterinary Parasitology, 186, 70–78. 22154968 10.1016/j.vetpar.2011.11.048

[eva13294-bib-0032] Kemper, K. E. , Emery, D. L. , Bishop, S. C. , Oddy, H. , Hayes, B. J. , Dominik, S. , Henshall, J. M. , & Goddard, M. E. (2011). The distribution of SNP marker effects for faecal worm egg count in sheep, and the feasibility of using these markers to predict genetic merit for resistance to worm infections. Genetics Research (Cambridge Core), 93(3), 203–19. 10.1017/S0016672311000097 24725775

[eva13294-bib-0033] Khansari, D. N. , Murgo, A. J. , & Faith, R. E. (1990). Effects of stress on the immune system. Immunology Today, 11, 170–175. 10.1016/0167-5699(90)90069-L 2186751

[eva13294-bib-0034] Lazzaro, B. P. , & Little, T. J. (2009). Immunity in a variable world. Philosophical Transactions of the Royal Society B: Biological Sciences, 364, 15–26. 10.1098/rstb.2008.0141 PMC2666692 18926975

[eva13294-bib-0035] Legarra, A. , Croiseau, P. , Sanchez, M. P. , Teyssèdre, S. , Sallé, G. , Allais, S. , & Elsen, J.‐M. (2015). A comparison of methods for whole‐genome QTL mapping using dense markers in four livestock species. Genetics, Selection, Evolution: GSE, 47, 6. 10.1186/s12711-015-0087-7 PMC4324410 25885597

[eva13294-bib-0036] Lynch, M. , & Walsh, B. (1998). Genetic x Environment interaction. In Genetics and analysis of quantitative traits. Sinauer Associates Inc.

[eva13294-bib-0037] McRae, K. M. , McEwan, J. C. , Dodds, K. G. , & Gemmell, N. J. (2014). Signatures of selection in sheep bred for resistance or susceptibility to gastrointestinal nematodes. BMC Genomics, 15, 637. 10.1186/1471-2164-15-637 25074012 PMC4124167

[eva13294-bib-0038] Minton, J. E. , & Blecha, F. (1990). Effect of acute stressors on endocrinological and immunological functions in lambs. Journal of Animal Science, 68, 3145–3151. 10.2527/1990.68103145x 2174846

[eva13294-bib-0039] Misztal, I. , Tsuruta, S. , Strabel, T. , Auvray, B. , Druet, T. , & Lee, D. (2002). BLUPF90 and related programs (BGF90). In Proceedings of the 7th world congress on genetics applied to livestock production (Vol. 33, pp. 743–744).

[eva13294-bib-0040] Ngere, L. , Burke, J. M. , Morgan, J. L. M. , Miller, J. E. , & Notter, D. R. (2018). Genetic parameters for fecal egg counts and their relationship with body weights in Katahdin lambs. Journal of Animal Science, 96, 1590–1599. 10.1093/jas/sky064 29635633 PMC6140914

[eva13294-bib-0041] O’Connor, L. J. , Walkden‐Brown, S. W. , & Kahn, L. P. (2006). Ecology of the free‐living stages of major trichostrongylid parasites of sheep. Veterinary Parasitology, 142, 1–15. 10.1016/j.vetpar.2006.08.035 17011129

[eva13294-bib-0042] Padgett, D. A. , & Glaser, R. (2003). How stress influences the immune response. Trends in Immunology, 24, 444–448. 10.1016/S1471-4906(03)00173-X 12909458

[eva13294-bib-0043] Pinheiro, J. , Bates, D. , Debroy, S. , & Sarkar, D. (2019). nlme: Linear and Nonlinear Mixed Effects Models (Version R package version 3.1‐140).

[eva13294-bib-0044] Pollott, G. E. , & Greeff, J. C. (2004). Genotype x environment interactions and genetic parameters for fecal egg count and production traits of Merino sheep. Journal of Animal Science, 82, 2840–2851. 15484934 10.2527/2004.82102840x

[eva13294-bib-0045] Pollott, G. E. , Karlsson, L. J. E. , Eady, S. , & Greeff, J. C. (2004). Genetic parameters for indicators of host resistance to parasites from weaning to hogget age in Merino sheep1. Journal of Animal Science, 82, 2852–2864. 10.2527/2004.82102852x 15484935

[eva13294-bib-0046] Proudfoot, K. , & Habing, G. (2015). Social stress as a cause of diseases in farm animals: Current knowledge and future directions. The Veterinary Journal, 206, 15–21. 26160470 10.1016/j.tvjl.2015.05.024

[eva13294-bib-0047] R Core Team (2016). R: A Language and Environment for Statistical Computing. R Foundation for Statistical Computing.

[eva13294-bib-0048] Riggio, V. , Pong‐Wong, R. , Sallé, G. , Usai, M. G. , Casu, S. , Moreno, C. R. , Matika, O. , & Bishop, S. C. (2014). A joint analysis to identify loci underlying variation in nematode resistance in three European sheep populations. Journal of Animal Breeding and Genetics, 131, 426–436. 10.1111/jbg.12071 24397290 PMC4258091

[eva13294-bib-0049] Robinson, M. R. , Wilson, A. J. , Pilkington, J. G. , Clutton‐Brock, T. H. , Pemberton, J. M. , & Kruuk, L. E. B. (2009). The impact of environmental heterogeneity on genetic architecture in a wild population of Soay sheep. Genetics, 181, 1639–1648. 10.1534/genetics.108.086801 19204380 PMC2666526

[eva13294-bib-0050] Russell, E. , Koren, G. , Rieder, M. , & Van Uum, S. (2012). Hair cortisol as a biological marker of chronic stress: Current status, future directions and unanswered questions. Psychoneuroendocrinology, 37, 589–601. 21974976 10.1016/j.psyneuen.2011.09.009

[eva13294-bib-0051] Sallé, G. , Moreno, C. , Boitard, S. , Ruesche, J. , Tircazes-Secula, A. , Bouvier, F. , Aletru, M. , Weisbecker, J.-L. , Prévot, F. , Bergeaud, J.-P. , Trumel, C. , Grisez, C. , Liénard, E. , & Jacquiet, P. (2014). Functional investigation of a QTL affecting resistance to *Haemonchus contortus* in sheep. Veterinary Research, 45, 68. 10.1186/1297-9716-45-68 24939584 PMC4077151

[eva13294-bib-0052] Sallé, G. , Jacquiet, P. , Gruner, L. , Cortet, J. , Sauvé, C. , Prévot, F. , & Moreno, C. R. (2012). A genome scan for QTL affecting resistance to *Haemonchus contortus* in sheep. Journal of Animal Science, 90, 4690–4705. 22767094 10.2527/jas.2012-5121

[eva13294-bib-0053] Seppälä, O. , & Jokela, J. (2016). Do coinfections maintain genetic variation in parasites? Trends in Parasitology, 32, 930–938. 10.1016/j.pt.2016.08.010 27614425

[eva13294-bib-0054] Smith, J. A. , Wilson, K. , Pilkington, J. G. , & Pemberton, J. M. (1999). Heritable variation in resistance to gastro‐intestinal nematodes in an unmanaged mammal population. Proceedings. Biological Sciences, 266, 1283–1290. 10418164 10.1098/rspb.1999.0776 PMC1690063

[eva13294-bib-0055] Sparks, A. M. , Hayward, A. D. , Watt, K. , Pilkington, J. G. , Pemberton, J. M. , Johnston, S. E. , & Nussey, D. H. (2020). Maternally derived anti‐helminth antibodies predict offspring survival in a wild mammal. Proceedings of the Royal Society B: Biological Sciences, 287, 20201931. 10.1098/rspb.2020.1931 PMC7739501 33234082

[eva13294-bib-0056] Sparks, A. M. , Watt, K. , Sinclair, R. , Pilkington, J. G. , Pemberton, J. M. , McNeilly, T. N. , Nussey, D. H. , & Johnston, S. E. (2019). The genetic architecture of helminth‐specific immune responses in a wild population of Soay sheep (*Ovis aries*). PLoS Genetics, 15, e1008461. 10.1371/journal.pgen.1008461 31697674 PMC6863570

[eva13294-bib-0057] Terefe, G. , Lacroux, C. , Andreoletti, O. , Grisez, C. , Prevot, F. , Bergeaud, J. P. , Penicaud, J. , Rouillon, V. , Gruner, L. , Brunel, J. C. , Francois, D. , Bouix, J. , Dorchies, P. , & Jacquiet, P. (2007). Immune response to *Haemonchus contortus* infection in susceptible (INRA 401) and resistant (Barbados Black Belly) breeds of lambs. Parasite Immunology, 29, 415–424. 10.1111/j.1365-3024.2007.00958.x 17650183

[eva13294-bib-0058] Terlouw, E. M. , Lawrence, A. B. , Ladewig, J. , De Passille, A. M. , Rushen, J. , & Schouten, W. G. (1991). Relationship between plasma cortisol and stereotypic activities in pigs. Behavioural Processes, 25, 133–153. 10.1016/0376-6357(91)90016-S 24923973

[eva13294-bib-0059] VanRaden, P. M. (2008). Efficient methods to compute genomic predictions. Journal of Dairy Science, 91, 4414–4423. 10.3168/jds.2007-0980 18946147

[eva13294-bib-0060] Verdú, J. R. , Lobo, J. M. , Sánchez‐Piñero, F. , Gallego, B. , Numa, C. , Lumaret, J.‐P. , & Durán, J. (2018). Ivermectin residues disrupt dung beetle diversity, soil properties and ecosystem functioning: An interdisciplinary field study. The Science of the Total Environment, 618, 219–228. 29128770 10.1016/j.scitotenv.2017.10.331

[eva13294-bib-0061] Woolaston, R. R. (1992). Selection of Merino sheep for increased and decreased resistance to *Haemonchus contortus*: Peri‐parturient effects on faecal egg counts. International Journal for Parasitology, 22, 947–953. 1459789 10.1016/0020-7519(92)90052-m

[eva13294-bib-0062] Woolaston, R. R. , Barger, I. A. , & Piper, L. R. (1990). Response to helminth infection of sheep selected for resistance to *Haemonchus contortus* . International Journal for Parasitology, 20, 1015–1018. 10.1016/0020-7519(90)90043-M 2074125

[eva13294-bib-0063] Yang, J. , Benyamin, B. , McEvoy, B. P. , Gordon, S. , Henders, A. K. , Nyholt, D. R. , Madden, P. A. , Heath, A. C. , Martin, N. G. , Montgomery, G. W. , Goddard, M. E. , & Visscher, P. M. (2010). Common SNPs explain a large proportion of the heritability for human height. Nature Genetics, 42, 565–569. 10.1038/ng.608 20562875 PMC3232052

[eva13294-bib-0064] Zhang, R. , Liu, F. , Hunt, P. , Li, C. , Zhang, L. , Ingham, A. , & Li, R. W. (2019). Transcriptome analysis unraveled potential mechanisms of resistance to *Haemonchus contortus* infection in Merino sheep populations bred for parasite resistance. Veterinary Research, 50. 10.1186/s13567-019-0622-6 PMC6345051 30678719

