# Supplementary material for: Genetic × environment variation in sheep lines bred for divergent resistance to strongyle infection
Source: Evol Appl. 2021 Sep 15;14(11):2591–602. doi: 10.1111/eva.13294 (PMC8591325; doi:10.1111/eva.13294)
Supplement: Supplementary file 1 — Supplementary Material [file EVA-14-2591-s002.pdf]

Supplementary Technical Note 1. Divergent sheep line creation 2

Supplementary Figure 1. QQ-plot of the association between genotyped SNPs and faecal egg count at first and second infection 7

Supplementary Figure 2. Offspring to midparent Faecal Egg Count measured following *H. contortus* infection 8

Supplementary technical note 2 : Changes in behavioural reactivity and standing-lying behaviour following chronic stress exposure 9

1. Measures of behavioural reactivity and standing-lying behaviour 9
2. Results 10

Supplementary Figure 3. Behaviour trajectory across experimental sheep groups 10

Supplementary Table 1. SNP metadata 12

Supplementary Table 2. Estimated contrasts and significance for considered fixed effects in implemented analyses 12

Supplementary Figure 4. Environmental effect on resistance potential varies across sire families 13

Supplementary Technical Note 1. Divergent sheep line creation

In November 2013, 277 Romane lambs were experimentally challenged by two successive infections with *Haemonchus contortus*. Lambs received an ivermectin treatment 30 days after the first infection and a two-week recovery period was applied before the second infection took place. Faecal egg Count and Packed Cell Volume was measured just before the infection, 24, and 30 days post-infection. In total, 241 individuals were phenotyped on both occasions (Table 1).

Genotyping was performed for 274 of these lambs with a set of 1,000 SNPs, including markers in 8 QTL regions previously detected in a backcross between Romane and Black Belly breeds (Sallé et al., 2012) and in two other European sheep populations (Riggio et al., 2014). Sires (n = 37) were also genotyped for the 1,000 SNPs to assign missing sire-offspring kinship in 216 lambs. The total pedigree considered in the following analyses included 3 generations and 2,572 individuals.

Table 1: Number of animal by sex and Faecal Egg Count measures among the 274-candidate animals

	Males	Females	Total
Phenotyped at 1st infection	133	122	255
Phenotyped at 2nd infection	132	125	257
Phenotyped in both infections	121	120	241

The founder individuals mated to create the first generation of divergent individuals (G1) were selected among 274 animals with phenotype and genotype information. Their breeding value was estimated following a single-step Best Linear Unbiased Prediction (BLUP) approach that uses both the genomic and pedigree information (with equal weights).

Table 2: Genetic information associated with sires used to create G1 lambs

Selected sire	Sire Status	Ewe	Sire	Sire gBV	Obtained G1 offsprings
20000132336	R	20000120766	20000120424	-2.10	51
20000132471	R	20000120075	20000120140	-1.21	39
20000132453	R	20000121413	0	-1.20	46
20000132361	S	20000120399	20000120375	1.07	41
20000132497	S	20000121045	20000120985	1.18	44
20000132550	S	20000120415	20000120985	1.30	13

Individuals with most extreme breeding values at first and second infection were selected, yielding 3 sires at each extreme end of the phenotypic range (Table 2). Of note, two of the susceptible sires were half-sibs hence reducing the sampling effort (lower *Ne*) of available genetic variability in the susceptible lineage (Falconer and Mackay, 1996). In ewes, the selection intensity was lower, *i.e.* the median breeding values was chosen as a cut-off, to ensure enough G1 lambs would be produced.

In March 2015, 236 G1 lambs were born (Table 3), out of which 80 individuals were needed for experimental groups. Among the 236 born lambs, 180 individuals were selected according to their expected breeding values (average of their parents genomic breeding values across 1st and 2nd infection) for genotyping using the same 1000-SNP panel. Their genomic breeding values were estimated using the G0 founder population as a reference panel. Based on the estimated genetic merit, 91 G1 individuals (43 males & 48 females) were selected to ensure a minimum difference of 0.5 standard deviation between the genomic breeding values of both lines and 1 standard deviation between their pedigree-based breeding values. For the first experimental trial (summer 2015), 87 individual lambs were ultimately enrolled to measure genotype x environment interactions mediated by chronic stress.

Table 3. number of animal by sex, line and stress conditions

	R males	R females	S males	S females	Total
Chronic stress	10	13	11	10	44
Enrichment	11	12	10	10	43
Total	21	25	21	20	87

A second generation of lambs was subsequently created (G2). The most extreme G1 lambs were chosen to retain 3 sires within each line (Table 4). To increase the genetic gain in females, 82 females were selected from both G0 and G1 ewes, ensuring 2 standard deviations between the average expected breeding values of the two lines. Controlled matings were performed between individuals with limited kinship ensuring an expected inbreeding coefficient in the G2 lambs lower than 0.04. Lambing took place in 55 out of the 82 selected ewes and generated 111 offsprings, 80 of which were selected according to the expected breeding values (average of their parents genetic merits ; Table 5).

Table 4. List of sires used to create the second generation G2

Sire of G2	Grand Sires of G2	Status
2000152347	20000132336	R
2000152365	20000132471	R
2000152226	20000132453	R
2000152258	20000132361	S
2000152337	20000132497	S
2000152434	20000132550	S

Table 5: Number of males and females by lines

Line	Males	Females	Total
R	31	23	54
S	26	31	57
total	57	54	111

Diagram of the selection procedure

Generation G0:

Birth &Phenotyping :

	males	females
Animal #	123	120
$\mu_{OPG} \pm \sigma_{OPG}$	9921±5000	9002±6352
μ_{uOPG}	0±1	0±1

Selection on u_{OPG} for mating :

	males		females	
	R	S	R	S
Selection rate (animal #)	2%(3)	2%(3)	47%(55)	53%(63)
μ_{uOPG}	-2.07	+1.64	-0.84	+0.87

Mating minimising inbreeding

Generation G1

Birth :

	males		females	
	R	S	R	S
Animal #	65	50	49	49
μ_{uOPG_parent}	-1.41	1.15	-1.42	1.14

Selection on u_{OPG_parent} for phenotyping :

	males		females	
	R	S	R	S
Selection rate (animal #)	34%(22)	44% (22)	49% (24)	39% (19)
$\mu_{OPG} \pm \sigma_{OPG}$	438±567	2514±1968	335±910	3268±2141
μ_{uOPG}	-2.8	2.7	-3.0	3.1

Selection on u_{OPG} for mating :

Male selection within sire family

Female selection within the generations 0 & 1

sexe	males		females			
Line origine	R	S	G1		G0	
	G1	G1	R	S	R	S
Selection rate (animal #)	5% (3)	5%(3)	96%(23)	100%(19)	35%(19)	30% (19)
μ_{uOPG}	-3.2	3.5	-3.2	3.2	-1.8	2.4

Mating minimising inbreeding

Generation G2

Birth :

	males		females	
	R	S	R	S
Animal #	31	27	25	31
μ_{uOPG_parent}	-2.9	3.2	-3.0	3.2

Selection on u_{OPG_parent} for phenotyping :

	males		females	
	R	S	R	S
Selection rate	65% (20)	70% (19)	76% (19)	65% (20)
$\mu_{OPG} \pm \sigma_{OPG}$	505±444	1670±1337	447±627	1350±1450
μ_{uOPG}				

Supplementary Figure 1. QQ-plot of the association between genotyped SNPs and faecal egg count at first and second infection

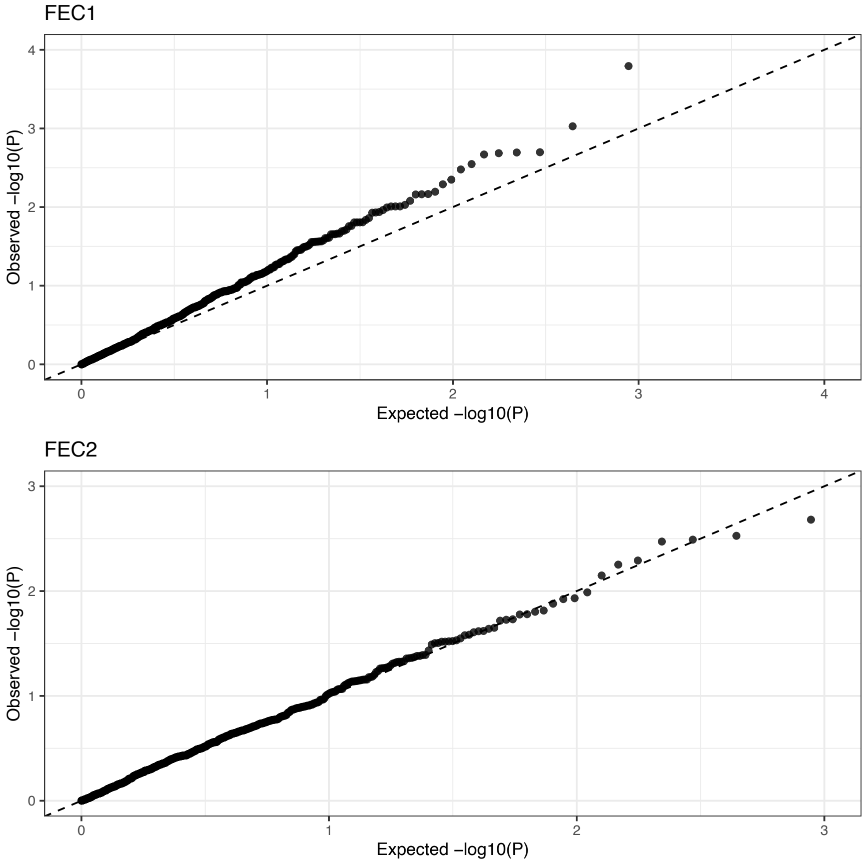

The figure represents the relationship between expected and observed P-values (represented as -log10(P)) following association between faecal egg count at first (FEC1) or second infection (FEC2) and 898 filtered SNPs located within 8 QTL regions in the G0 population. Associated genomic inflation factors were 1.19 and 1.14 for FEC1 and FEC2 respectively.

Supplementary Figure 2. Offspring to midparent Faecal Egg Count measured following *H. contortus* infection

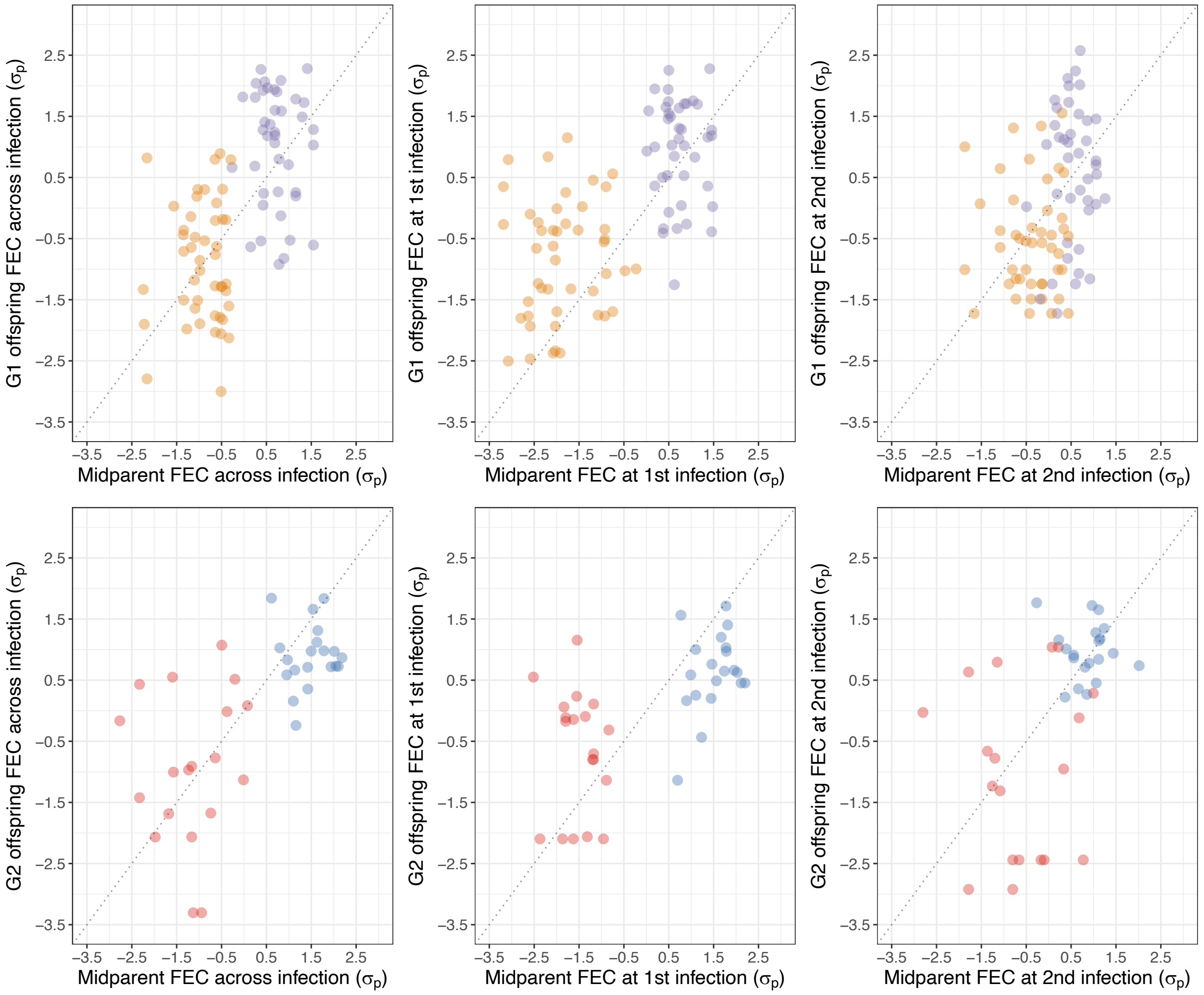

The figure represents average Faecal Egg Count (FEC) of *H. contortus* infected lambs, plotted against their respective midparent values (mean phenotype values of respective sire and dam). Top and bottom panels correspond to data gathered in the first (G1) and second (G2) generation of divergence respectively, with dot colors matching genetic lines (orange and red for resistant individuals in G1 and G2 respectively). For each infection, FEC were averaged across two measures recorded at 24 and 30 days after 1st (middle) or 2nd (right) infection or across infections (left), normalized (fourth-root transformed) and corrected for environmental effects. Resulting corrected FEC were then standardized (mean centered and reduced to G0 standard deviation unit, σ_p) for the sake of comparison across generations. Dotted line materializes complete genetic response (heritability of 1).

Supplementary technical note 2 : Changes in behavioural reactivity and standing-lying behaviour following chronic stress exposure

1. Measures of behavioural reactivity and standing-lying behaviour

Measurements focused on behavioural reactivity and standing-lying behaviour that were recorded before the onset of behavioural treatments, five weeks later, and 14 days after the first and second infections. Additional recordings of standing-lying behaviour were performed on the same day and just before experimental infection took place.

Behavioural reactivity was measured by an arena test (2 m x 7 m dirt floor with 2 m high solid walls and 7 equal-sized areas delimited by lines on the floor) that evaluate social attractiveness of two lambs for their flock-mates (n = 3, kept behind a wire mesh) or for a standing human being. The first phase of the test (1 min) evaluated reaction to novelty and social isolation (measured by locomotion, *i.e.* number of squares entered, and vigilance, *i.e.* head in upright position and ears perpendicular to the head). Tested lambs were first isolated from their flock-mates (hidden behind a curtain) for 1 min before the curtain was lifted to allow social proximity between tested lambs and their mates. The second phase consisted in an operator entering the arena and standing in front of the wire mesh for 1 min to measure lamb social attraction and reaction to a stationary person (locomotion, vigilance, bleat, physical contact with their flock-mates and with the operator). Lamb behaviour was recorded with a camera (Sony SPT-MC128CE, Sony Corp., Tokyo, Japan) and video recorder (Sony SVT-1000P, Sony Corp., Tokyo, Japan). Vocalisations were recorded directly by an observer hidden from lambs.

Standing-lying behaviour was recorded on females with an accelerometer (HOBO® Pendant®) attached with a cohesive bandage to the lateral side of the hind leg (left or right in half the lambs each). The accelerometer was positioned so that the x-axis was vertical and towards the ground, and the y-axis parallel to the ground and towards the rear of the animal.

2. Results

Average values recorded in resistant and susceptible lines either before or after chronic stress are presented in supplementary Figure 3 and detailed statistics are provided under supplementary Table 2.

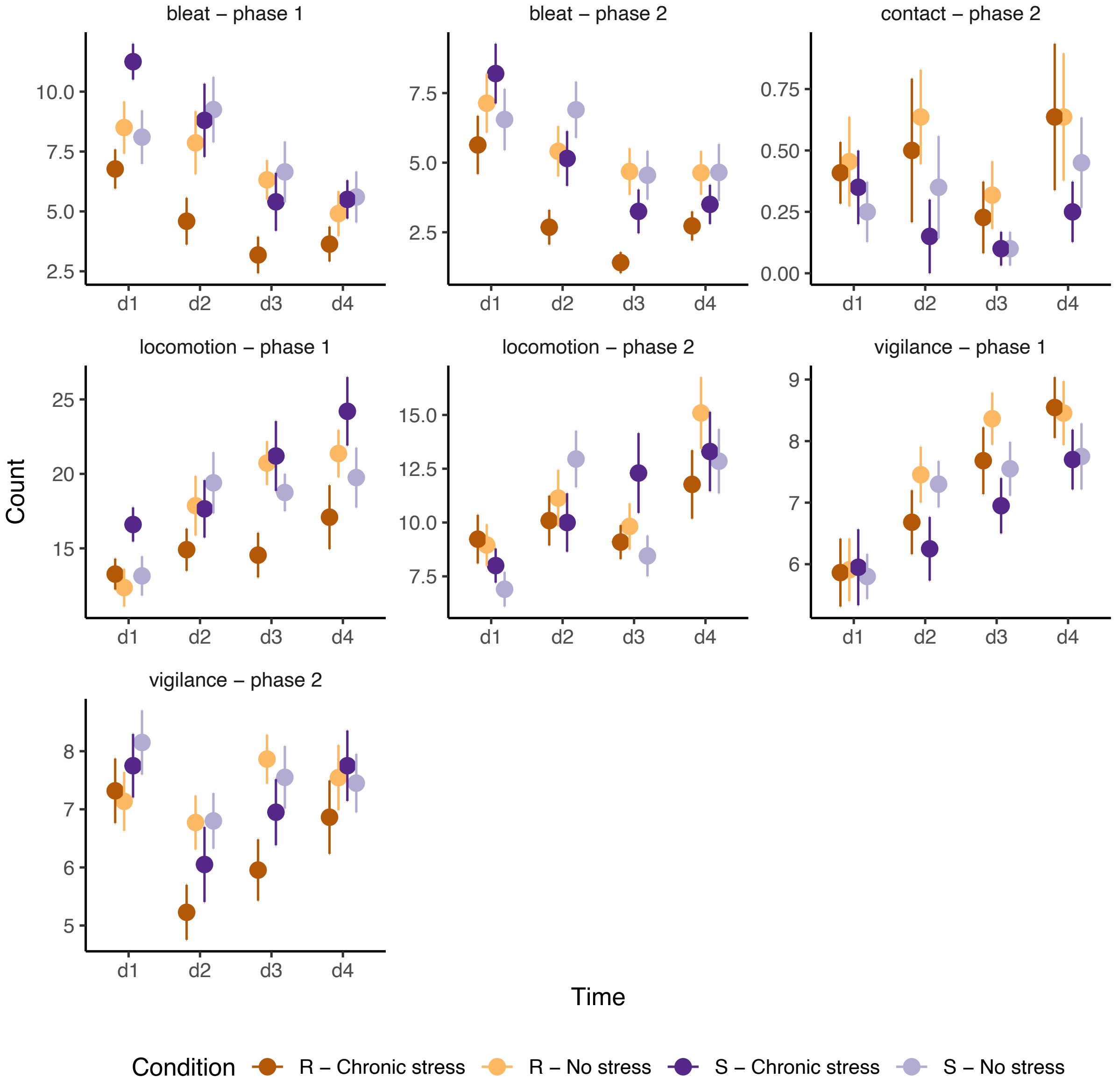

Supplementary Figure 3. Behaviour trajectory across experimental sheep groups

Average behaviour trait values recorded in resistant (R; dark and light brown) and susceptible (S; dark and light purple) sheep is plotted across time points with corresponding standard errors. Time points correspond to before behaviour treatment, after treatment before infection, 14 days after the first infection, and 14 days after the second infection.

Before chronic stress was applied, female lambs from the control group spent more time standing than female lambs in the other group (628 vs 578 min/day ; $F_{1,36} = 6.47$, $P = 0.01$, supplementary Table 2). This variable was hence not considered further. No significant difference was found in other behavioural data between both treatment groups at that time (supplementary Table 2).

Following exposure to chronic stress for five weeks and before any infection took place, lambs displayed altered behaviour (supplementary Figure 3, supplementary Table 2). They bleated less in phase 2 of the arena test throughout the experiment (2.35 count difference, $F_{1,79} = 6.09$, $P = 0.02$, supplementary Table 2). They also expressed less vigilance (phase 2) than their counterparts facing control conditions throughout the experiment (0.69 count difference, $F_{1,80} = 5.06$, $P = 0.03$, supplementary Table 2).

Supplementary Table 1. SNP metadata

See attached file.

SNP positions (OAR: chromosome, position in bp) and identifiers (SNPid) are listed for the 828 pruned SNPs considered for association study in the G0 population.

Supplementary Table 2. Estimated contrasts and significance for considered fixed effects in implemented analyses

See attached file.

Table provides output summary for every reported modeling of response to selection, asymmetry of that response, weight trajectory, behaviour data, genotype x environment interaction across or within sire families. For every summary, contrasts between reference and other factor levels are given with respective standard errors (s.e.), t-test values and *P*-value. Any significant difference ($P < 0.05$) is highlighted in green.

Supplementary Figure 4. Environmental effect on resistance potential varies across sire families

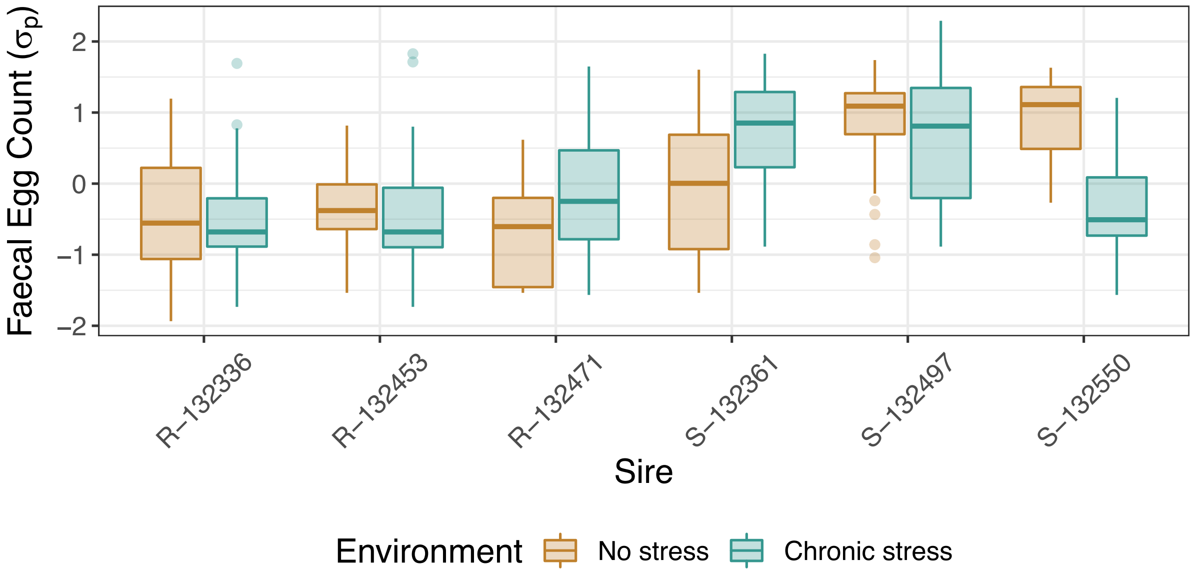

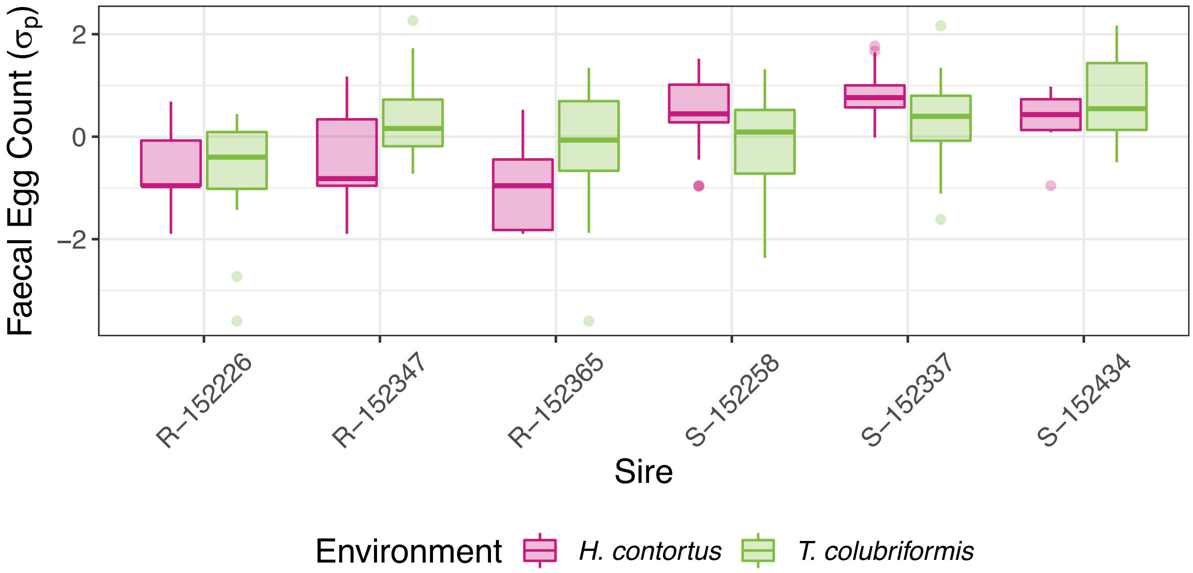

This figure represents the within-sire family variation in Faecal Egg Count (FEC, given in phenotypic standard deviation σ_p) across environmental conditions. FEC were normalized (fourth-root transformation) and then mean centered and scaled to unit standard deviation within each experimental block for the sake of comparison across conditions. First sire name letter indicates the divergent line it belongs to (R, resistant or S, susceptible). The picture indicates that the magnitude of G x E interaction varies among sires across stress conditions (top panel): sires S-132361 and S-132550 had their progenies showing higher or lower susceptibility under chronic stress respectively. The trend in resistance was conserved across GIN species in every sire family (bottom panel).

References

Riggio, V., Pong-Wong, R., Sallé, G., Usai, M. G., Casu, S., Moreno, C. R., … Bishop, S. C. (2014). A joint analysis to identify loci underlying variation in nematode resistance in three European sheep populations. *J Anim Breed Genet*, *131*, 426-436.

Sallé, G., Jacquiet, P., Gruner, L., Cortet J, Sauvé, C., Prévot, F., … Moreno, C. R. (2012). A genome scan for QTL affecting resistance to Haemonchus contortus in sheep. *J Anim Sci*, *90*, 4690-4705.
